# Hematological shift in goat kids naturally devoid of prion protein

**DOI:** 10.3389/fcell.2015.00044

**Published:** 2015-07-08

**Authors:** Malin R. Reiten, Maren K. Bakkebø, Hege Brun-Hansen, Anna M. Lewandowska-Sabat, Ingrid Olsaker, Michael A. Tranulis, Arild Espenes, Preben Boysen

**Affiliations:** Faculty of Veterinary Medicine and Biosciences, Norwegian University of Life SciencesOslo, Norway

**Keywords:** cellular prion protein, PrP^C^, hematology, hematopoiesis, phagocytosis, T-cell proliferation

## Abstract

The physiological role of the cellular prion protein (PrP^C^) is incompletely understood. The expression of PrP^C^ in hematopoietic stem cells and immune cells suggests a role in the development of these cells, and in PrP^C^ knockout animals altered immune cell proliferation and phagocytic function have been observed. Recently, a spontaneous nonsense mutation at codon 32 in the *PRNP* gene in goats of the Norwegian Dairy breed was discovered, rendering homozygous animals devoid of PrP^C^. Here we report hematological and immunological analyses of homozygous goat kids lacking PrP^C^ (*PRNP^Ter/Ter^*) compared to heterozygous (*PRNP*^+/Ter^) and normal (*PRNP*^+/+^) kids. Levels of cell surface PrP^C^ and *PRNP* mRNA in peripheral blood mononuclear cells (PBMCs) correlated well and were very low in *PRNP*^Ter/Ter^, intermediate in *PRNP*^+/Ter^ and high in *PRNP*^+/+^ kids. The *PRNP*^Ter/Ter^ animals had a shift in blood cell composition with an elevated number of red blood cells (RBCs) and a tendency toward a smaller mean RBC volume (*P* = 0.08) and an increased number of neutrophils (*P* = 0.068), all values within the reference ranges. Morphological investigations of blood smears and bone marrow imprints did not reveal irregularities. Studies of relative composition of PBMCs, phagocytic ability of monocytes and T-cell proliferation revealed no significant differences between the genotypes. Our data suggest that PrP^C^ has a role in bone marrow physiology and warrant further studies of PrP^C^ in erythroid and immune cell progenitors as well as differentiated effector cells also under stressful conditions. Altogether, this genetically unmanipulated PrP^C^-free animal model represents a unique opportunity to unveil the enigmatic physiology and function of PrP^C^.

## Introduction

The cellular prion protein (PrP^C^) was first described as the substrate for PrP scrapie (PrP^Sc^) (Prusiner, 1982; Brandner et al., 1996a,b), a misfolded and aggregation-prone form of the protein detected in brain tissue of animals diagnosed with transmissible spongiform encephalopathies, now often called prion diseases. These are fatal neurodegenerative diseases occurring naturally in humans and ruminants and include Creutzfeldt-Jakob disease, bovine spongiform encephalopathy, scrapie, and chronic wasting disease. The process of template-directed self-replication of PrP^Sc^ constitutes the core of the “protein only” hypothesis (Prusiner et al., 1998), stating that the prion agent consists solely of misfolded PrP^C^ conformers, and in accordance with this, PrP^C^ knock-out (KO) mice do not replicate prions, nor do they develop prion disease (Bueler et al., 1992).

PrP^C^ is a highly conserved GPI-anchored protein (Steele et al., 2007) expressed abundantly in the central nervous system (CNS) (Kretzschmar et al., 1986), but also at lower levels in many other cells like hematopoietic (Zhang et al., 2006) and embryonic stem cells (Miranda et al., 2011), immune cells (Isaacs et al., 2006) and various epithelial cell types (Horiuchi et al., 1995; Ford et al., 2002), suggesting that PrP^C^ might have important functions within these cell types (Bendheim et al., 1992). In view of this, it was puzzling that PrP^C^ KO mice developed normally without overt phenotypic abnormalities (Bueler et al., 1992; Manson et al., 1994). Subsequent analyses have; however, revealed various phenotypes such as altered circadian rhythms (Tobler et al., 1996), behavior abnormalities (Roesler et al., 1999; Massimino et al., 2013), increased susceptibility to oxidative stress (Wong et al., 2001), and increased excitability of neurons (Khosravani et al., 2008).

In bone marrow, PrP^C^ is expressed in long-term hematopoietic stem cells (Dodelet and Cashman, 1998; Zhang et al., 2006) and may contribute to maintenance of stem cell properties, since bone marrow stem cells derived from PrP^C^ KO mice, contrary to similar cells from wild-type mice, fail to repopulate the bone marrow of irradiated recipient mice, especially after serial transplantations (Zhang et al., 2006). Interestingly, the PrP^C^ expression in immune cells is regulated according to their lineage fate. The observation that PrP^C^ expression is maintained at a high level in mononuclear cell precursors and downregulated in granulocytic and erythroid cells during their maturation in the bone marrow suggests that PrP^C^ plays a role in the dynamic development of these cells (Dodelet and Cashman, 1998). PrP^C^ is upregulated in activated T lymphocytes (Mabbott et al., 1997) and neutrophils (Mariante et al., 2012) and has been suggested to act as a signaling molecule in the activation of immune cells (Mattei et al., 2004; Krebs et al., 2006), but the definite role of PrP^C^ in these processes remains unclear.

Whether PrP^C^ modulates macrophage functions, in particular phagocytic capacity is controversial. Peritoneal macrophages devoid of PrP^C^ displayed increased phagocytosis toward apoptotic cells in comparison with wild-type macrophages (De Almeida et al., 2005), suggesting that PrP^C^ acts as a negative modulator of phagocytosis. Similar results were recently reported in a study using primary cell culture of bone marrow-derived macrophages from ZrchI type PrP^C^ KO mice in comparison with similar cells derived from C57BL/6 mice (Wang et al., 2014), where the PrP^C^-depleted macrophages displayed increased phagocytic capacity toward fluorescently labeled *E. coli*, enhanced phagosome maturation and cytokine expression. Uraki and co-workers; however, reached the opposite conclusion when using immortalized bone marrow-derived macrophages from ZrchI type PrP^C^ KO mice, observing that loss of PrP^C^ reduced phagocytic capacity toward fluorescent latex beads (Uraki et al., 2010).

Likewise, the proliferative response of T cells *in vitro* upon cytokine stimulation has been studied in cells from transgenic mice and cattle with and without PrP^C^. Murine studies demonstrated a reduced proliferative response (Mabbott et al., 1997) and altered cytokine profile (Bainbridge and Walker, 2005) in Concanavalin A (Con A)-stimulated T cells from PrP^C^ KO mice indicating a role for PrP^C^ in T-cell pathways leading to proliferation. Data from transgenic PrP^C^ KO cattle (Richt et al., 2007) revealed no differences in the proliferative response of T cells when stimulated with anti-CD3 antibody, Con A and phytohemagglutinin. A further clarification of PrP^C^'s roles in stem cell and immune cell maturation and function is desirable and will be helpful in understanding PrP^C^'s functions in general.

Recently, a unique line of otherwise healthy Norwegian dairy goats carrying a nonsense mutation at codon 32 (premature termination codon, Ter) in the *PRNP* reading frame was discovered (Benestad et al., 2012). The mutation results in an early termination of PrP^C^ synthesis and consequently animals homozygous for this mutation are devoid of PrP^C^. This is, to our knowledge, the first species identified that is naturally devoid of PrP^C^. These animals are genetically unmanipulated and therefore represent a unique spontaneous animal model to complement the transgenic models available for studies of PrP^C^ functions.

Here, we report results of hematological and immunological analyses of goat kids without PrP^C^ expression (*PRNP*^Ter/Ter^) compared to heterozygotes (*PRNP*^+/Ter^) and normal (*PRNP*^+/+^) goats.

## Methods

### Animals and sampling

The animals included in this study were of the Norwegian Dairy Goat Breed obtained from a research herd of around 100 animals at the Norwegian University of Life Sciences. Based on daily monitoring and enhanced health surveillance through a national goat health monitoring service, the health status of this flock is considered to be very good. Scrapie outbreaks have never occurred and there have been no cases of caprine arthritis encephalitis, Johne's disease or caseous lymphadenitis during the last 5 years (i.e., diseases subject to surveillance and control program in Norway) (Nagel-Alne et al., 2014). The entire flock was previously analyzed for *PRNP* genotypes (Benestad et al., 2012) and through selective breeding goat kids with the desired *PRNP*^Ter/Ter^ genotypes were retrieved. No animals died of disease during the observation time and all *PRNP*^Ter/Ter^ offspring developed normally during their first 6 months with no signs of behavioral or health problems. The Norwegian Animal Research Authority approved the protocol with reference to the Norwegian regulation on animal experimentation (FOR-1996-01-15-23, § 2) which is based upon the European Convention for the Protection of Vertebrate Animals used for Experimental and Other Scientific Purposes.

Blood was drawn from the jugular vein into heparinized tubes and tubes without anticoagulant from age matched goat kids at 3–4 weeks of age. For hematological and clinical chemistry analyses, goat kids carrying the genotypes *PRNP*^Ter/Ter^ (*n* = 8), *PRNP*^+/Ter^ (*n* = 16), and *PRNP*^+/+^ (*n* = 24) were included.

Hematological analyses were performed with an Advia® 2120 Hematology System using Advia 2120 MultiSpecies System Software and clinical chemistry analyses were performed with Advia 1800 Chemistry System (both from Siemens AG Healthcare Sector).

Peripheral blood mononuclear cells (PBMCs) were isolated from *PRNP*^Ter/Ter^ (*n* = 8) and *PRNP*^+/+^ (*n* = 8) goat kids by gradient centrifugation (Lymphoprep, Axis-Shield) at 1760 × g. Red blood cells (RBC) were lysed by brief exposure to sterile water prior to counting and trypan blue viability assessment using a Countess Automated Cell Counter (Life Technologies).

### Morphology

For morphological studies, two age-matched male kids, one *PRNP*^Ter/Ter^ and one *PRNP*^+/+^, were necropsied at 3 months of age. Macroscopic examinations were performed routinely. Fresh bone marrow imprints and smears were made and evaluated after staining with May Grünewald Giemsa. Histological slides were prepared from formalin fixed and paraffin-embedded tissues and stained with hematoxylin and eosin (HE) prior to examination by light microscopy.

### Real-time RT-PCR analysis

Total RNA was isolated from PBMCs using the Qiagen RNeasy mini plus kit (Qiagen) following the manufacturer's instructions. RNA concentration and purity was analyzed using NanoDrop-1000 Spectrophotometer (Thermo Fisher Scientific), and quality was assessed using RNA Nano Chips on an Agilent 2100 Bioanalyzer (both from Agilent Technologies). RNA was stored at −80°C. cDNA was synthesized using the SuperScript III Reverse Transcriptase, RNase Out, dNTP mix and Random Primers (all from Invitrogen, Life Technologies) at the following conditions: 5 min at 65°C, >1 min on ice, 5 min at 25°C, 1 h at 50°C, and 15 min at 70°C.

Quantitative-PCR was conducted with LightCycler 480 Sybr Green I Master mix (Roche), with *PRNP* as target gene (F: GTG GCT ACA TGC TGG GAA GT; R: AGC CTG GGA TTC TCT CTG GT) and glyceraldehyde 3-phosphate dehydrogenase (*GAPDH*) as reference gene (F: GGT TGT CTC CTG CGA CTT CA; R: TGG AAA TGT GTG GAG GTC GG). cDNA corresponding to 1 ng RNA was used per reaction. The samples had a total volume of 20 μl, and were run on a LightCycler 480 System (Roche). Conditions: 5 min at 95°C; 40 cycles of 10 s at 95°C, 10 s at 60°C, and 10 s at 72°C; and melting curve with 5 s at 95°C, 1 min at 65 and 97°C. Relative expression levels were calculated using an externally run standard curve for the PBMCs generated from one *PRNP*^+/+^ animal, run in duplicates, with one randomly selected *PRNP*^+/+^ as positive control (in-run).

### Flow cytometry for cell surface PrP and immune cell markers

Immunophenotyping in flow cytometry was performed as previously described (Olsen et al., 2013). Briefly, isolated PBMCs, or whole blood if indicated, were incubated with Fixable Yellow Dead Cell Stain Kit (Life Technologies, Thermo Fisher Scientific Inc.) followed by primary monoclonal antibodies (mAbs), brief incubation with 30% normal goat serum to block Fc-receptors, and finally fluorescence-labeled goat-anti-mouse secondary antibodies (see Supplementary Table 1). To detect the intracellular CD3 epitope, surface-labeled cells were permeabilized with Intracellular Fixation and Permeabilization Buffer Set (eBioscience, Affymetrix Inc.) according to the manufacturer's instructions. Labeled cells were analyzed in a Gallios flow cytometer and data were processed using Kaluza 1.2 software (both Beckman Coulter, Inc.). Cell gates were designed to select for single and viable mononuclear cells.

### Lymphocyte proliferation test

PBMCs were incubated in a flat-bottom 96 well plate for 72 h in complete medium with a density of 2 × 10^5^ cells per well. Cells were stimulated with Concanavalin A (ConA; Sigma-Aldrich) (200 μg/ml), recombinant ovine interleukin (IL)-2 (10,000 U/ml) (Connelley et al., 2011) or recombinant human (rh)IL-15 (Affymetrix/eBioscience) (25,000 U/ml). Unstimulated cells were used as controls. Each treatment was run in three to six parallels. Proliferation was measured as 24 h uptake of 3H-thymidine (Perkin-Elmer, Waltham, USA) as previously described (Storset et al., 2001) in counts per minute (CPM). Parallels outside ±50% of the median were excluded, and net proliferation (net CPM) was calculated as mean of stimulated cells minus mean of unstimulated cells.

### Isolation of monocytes and phagocytosis assays

CD14^+^ monocytes were positively selected from isolated PBMCs by anti-human CD14 MACS MicroBeads (Miltenyi Biotec GmbH, Bergisch Gladbach, Germany) using 10 μl beads per 10^7^ cells. The purity was consistently measured between 90 and 95% by flow cytometry using anti-CD14 mAbs (TUK4; IgG2a; AbD Serotec). CD14^+^ monocytes were seeded in a 96 well non-adherent plate (Corning Costar Ultra-Low Attachment multiwell plates) in complete medium [RPMI + 10% fetal calf serum (FCS) + 1% Penicillin/Streptomycin; Gibco] at 2 × 10^5^ cells per well and incubated for 24 h at 37°C and 5% CO_2_ in the presence of 5000 U/ml recombinant bovine granulocyte macrophage colony stimulating factor (rbGM-CSF) that had been expressed in 293T cells as described (Lund et al., 2012) based on a btGM-CSF plasmid (kindly provided by D. Werling, RVC, UK). The resulting activated monocytes were incubated with latex beads (FluoroSpheres 430/465, 20 μl corresponding to 4 × 10^6^ beads, 20 beads/cell, Life Technologies), pHrodo red *E. coli* or pHrodo red Zymosan (both from Life Technologies) in separate wells. Phagocytosis was terminated after 30 min of incubation (37°C, 5% CO_2_) by placing the plate on ice. The particle uptake was measured in a Gallios flow cytometer. The monocyte gate was adjusted to exclude free particles using cell-free particle-only samples as control.

To confirm that the particles had been phagocytized, monocytes were centrifuged onto slides at 1000 rpm for 5 min. The cells were fixed in acetone, blocked with goat serum and immunolabeled with mAb against human CD68 (Dako). The cytospots were incubated with secondary antibodies, Alexa 488 goat-anti-mouse IgG1 or Alexa 594 goat-anti-mouse IgG1, mounted in Prolong Gold Antifade reagent with DAPI (all from Life Technologies) and analyzed by standard fluorescence and confocal microscopy.

### Statistical analysis

All data was analyzed for statistical significance with the Mann-Whitney test and data reported with medians. A level of *P* < 0.05 was considered statistically significant and shown as ^*^ and ^**^ in figures. GraphPad prism version 6.04 software was used for statistical analyses.

## Results

### Expression of PrP^C^ in leukocytes correlates with genotype

**Figure 1:**
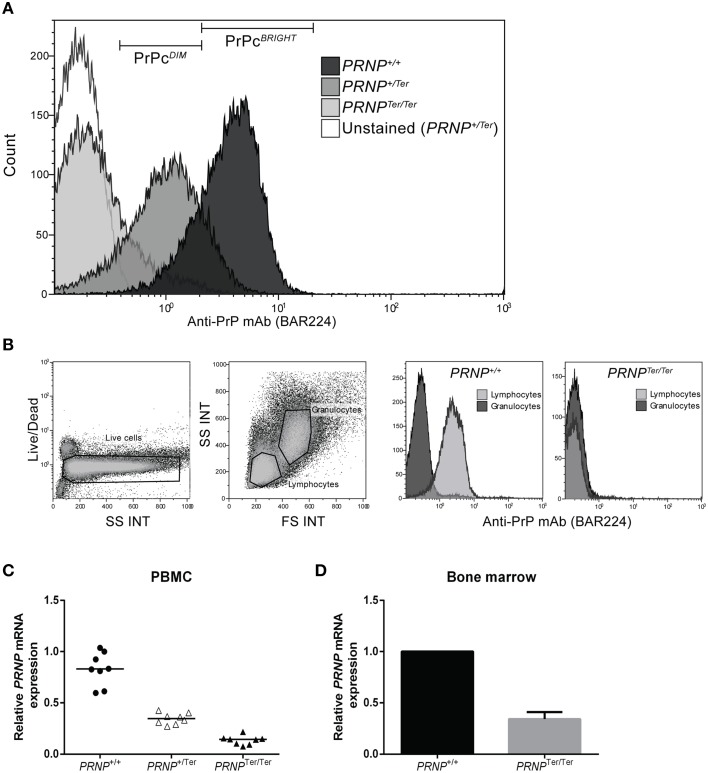
**(A–D)** Relative PrP^C^ expression and *PRNP* mRNA expression in PBMCs. **(A)** Representative plot of PrP^C^ expression on PBMCs is shown using anti-PrP^C^ mAb BAR224. **(B)** Whole blood analysis showing gating of live cells into granulocyte and lymphocyte populations based on FS and SS characteristics. The two rightmost plots show representative PrP^C^ expression on lymphocytes and granulocytes in *PRNP*^+/+^ (*n* = 6) and *PRNP*^Ter/Ter^ (*n* = 2) animals. **(C)** Relative *PRNP* mRNA expression in PBMCs (each genotype *n* = 8). **(D)** Relative *PRNP* mRNA expression in bone marrow (each genotype, *n* = 1).

The absence of PrP^C^ on PBMCs from *PRNP*^Ter/Ter^ goats was confirmed by three different anti-PrP^C^ mAbs (Figure 1A and data not shown). The fluorescence intensity of anti-PrP^C^-stained PBMCs showed that cells from *PRNP*^+/Ter^ goats expressed approximately half the density of surface PrP^C^ as compared to those from *PRNP^+/+^* goats. When co-staining for immune cell subpopulations, this pattern of PrP^C^ expression was similar in monocytes (CD14^+^), B cells (B-B2^+^), and T cells (CD3^+^) (data not shown). Anti-PrP^C^ mAbs staining of whole blood revealed that PrP^C^ was not expressed on the surface of granulocytes in goats of either genotype, while its presence was confirmed in the lymphocyte gate in *PRNP^+/+^* goats (Figure 1B).

To investigate whether reduced PrP^C^ levels in *PRNP*^+/Ter^ and *PRNP*^Ter/Ter^ animals would lead to compensatory upregulation of *PRNP* mRNA, relative mRNA expression levels in PBMCs and bone marrow were analyzed. As shown in Figures 1C,D, the *PRNP* mRNA expression level in PBMCs from *PRNP*^Ter/Ter^ animals was 16.8% of the expression in *PRNP*^+/+^ animals, whereas the expression level in *PRNP*^+/Ter^ PBMCs was 42.4% of the *PRNP*^+/+^ animals. In the bone marrow, the relative *PRNP* mRNA expression in the *PRNP*^Ter/Ter^ goat was 34% of the *PRNP*^+/+^ goat investigated (Figure 1D). Collectively, these data indicate that no compensatory mechanism is counteracting loss of PrP^C^ at *PRNP* mRNA expression level and that *PRNP*^Ter/Ter^-encoding mRNA is degraded.

### Red blood cell numbers are elevated in *PRNP*^Ter/Ter^ animals

To analyze whether reduced levels or complete lack of PrP^C^ influences the cellular or chemical composition of the blood, we performed hematological and clinical chemistry analyses of *PRNP*^Ter/Ter^, *PRNP*^+/Ter^ and *PRNP*^+/+^ goat kids between 3 and 4 weeks of age.

Clinical chemistry revealed a slightly, but significantly, lower magnesium level and an increased creatine level in *PRNP*^Ter/Ter^ animals compared to *PRNP*^+/+^, both well within the normal reference range (see Supplementary Table 2).

**Figure 2:**
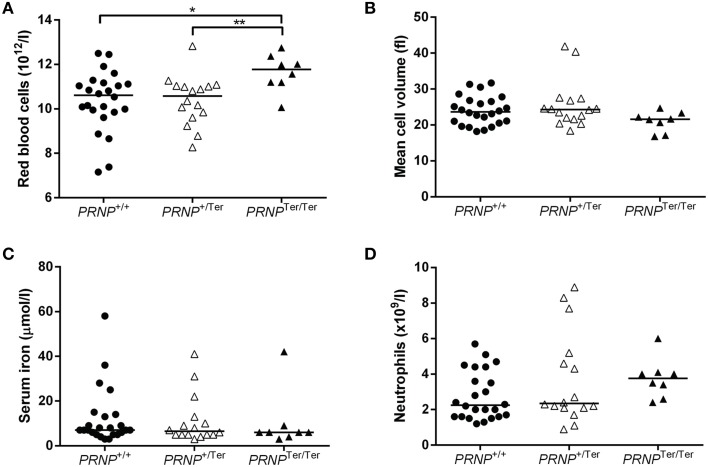
**Selected hematology parameters and serum iron values from 3 to 4 weeks old goat kids**. Dot plots showing **(A)** the number of RBCs, **(B)** the RBC mean cell volume, **(C)** the serum iron levels, and **(D)** absolute number of neutrophils in *PRNP*^Ter/Ter^ (*n* = 8), *PRNP*^+/Ter^ (*n* = 16), and *PNRP*^+/+^ (*n* = 24) animals. ^*^ and ^**^ indicate *P* < 0.05.

The number of RBCs was increased in *PRNP*^Ter/Ter^ goat kids (Table 1 and Figure 2A) as compared to the *PRNP*^+/+^ and *PRNP*^+/Ter^ groups. Mean cell volume of the RBCs (MCV) was not significantly different between the groups, although the distribution of values suggested a clear tendency of lower MCV in the *PRNP*^Ter/Ter^ animals compared to the *PRNP*^+/+^ and *PRNP*^+/Ter^ groups (Table 1 and Figure 2B).

**Table 1:** **Hematology results**.

	**Reference range**	**Median**			***P*-values**		
		**A: *PRNP*^+/+^**	**B: *PRNP*^+/Ter^**	**C: *PRNP*^Ter/Ter^**	**A vs. B**	**B vs. C**	**A vs. C**
White blood cells (× 10^9^/l)	4–16	7.5	7.4	8.65	0.75	0.73	0.34
Red blood cells (× 10^12^/l)	8–18	10.61	10.58	11.78	0.8	**0.006**	**0.007**
Hemoglobin (g/l)	75–125	66	67	67	0.51	0.87	0.82
Hematocrit (l/l)	0.22–0.38	0.25	0.26	0.25	0.51	0.8	0.92
Mean corpuscular volume (fl)	16–25	23.65	24.3	21.6	0.67	0.051	0.08
Mean corpuscular hemoglobin content (g/l)	320–370	267.5	262	270	0.51	0.11	0.058
Rdw (%)	23–35	53.8	53.1	52.2	0.99	0.62	0.76
Neutrophils (× 10^9^/l)	1.5–8	2.25	2.35	3.75	0.35	0.33	0.068
Lymphocytes (× 10^9^/l)	2–9	4.8	4.35	4.35	0.28	0.75	0.6
Monocytes (× 10^9^/l)	0–0.5	0.35	0.45	0.45	0.028	0.91	0.08

*P-values < 0.05 are shown in bold*.

The groups did not differ in hematocrit (HCT) values (Table 1), suggesting that the reduced RBC volume was compensated by an increased number of RBCs or vice versa. To investigate if differences in iron uptake and metabolism could have any influence on RBC number and MCV we investigated the iron levels in blood serum. However, iron levels did not differ between the genotypes (Figure 2C).

In blood smears from *PRNP*^Ter/Ter^ (*n* = 8) and *PRNP*^+/+^ (*n* = 24) animals a marked poikilocytosis was observed, as expected in young kids. The neutrophil granulocytes were mature and showed no signs of left shift in any of the genotypes. No reticulocytes were present in any of the smears (data not shown).

Within the leukocyte populations, there was a tendency of a higher neutrophil count in the *PRNP*^Ter/Ter^ animals as well as an increase in the monocyte number in *PRNP*^Ter/Ter^ and *PRNP*^+/Ter^ animals compared to the *PRNP*^+/+^ group (Table 1 and Figure 2D). The majority of the hematology values were within the general reference ranges for goats, except mean cell hemoglobin content (MCHC), red cell distribution width (RDW), and hemoglobin, where values fell below the presented reference range in all genotypes. However, breed-specific reference ranges were not available, and since these low values were present in all groups they would most likely be within normal ranges for rapidly growing goat kids of this breed.

**Figure 3:**
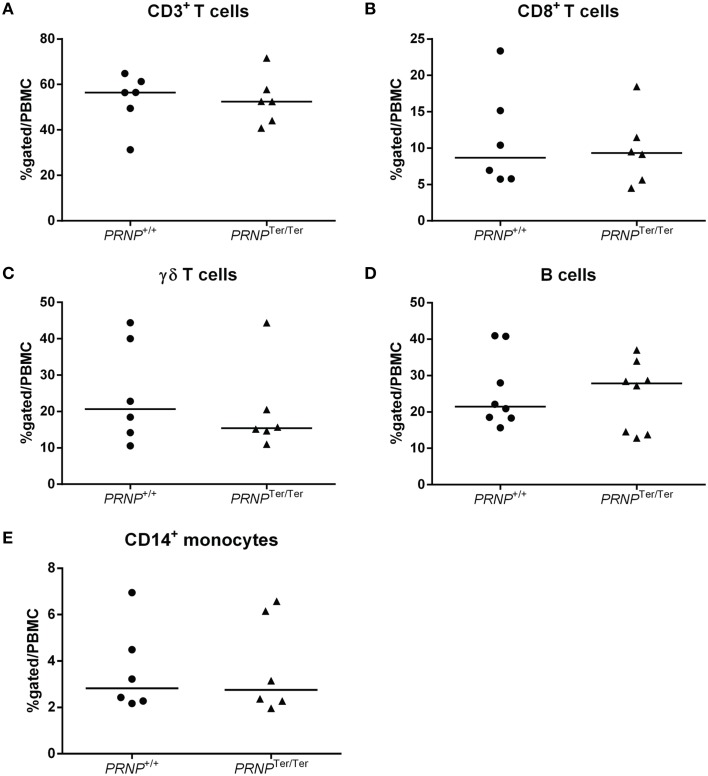
**Relative immune cell population sizes in 3–4 weeks old goat kids**. Flow cytometric analysis of **(A)** CD3^+^ T cells, **(B)** CD8^+^ T cells, **(C)** γδ T cells, **(D)** B cells, and **(E)** CD14^+^ monocytes out of the total PBMC population.

Observing that PrP^C^ was highly expressed in a range of immune cells in normal goats we investigated whether lack of PrP^C^ expression influenced the numbers of these cells in peripheral blood. The relative numbers of monocytes (CD14^+^), B cells (B-B2^+^), T cells (CD3^+^) as well as the gamma-delta (WC1^+^) and CD8^+^ subsets of T cells were quantified by flow cytometry (Figure 3). CD4^+^ T-cell labeling was excluded from the study due to methodological problems. No differences in numbers were revealed for any of these leukocyte subsets between the *PRNP*^+/+^ and *PRNP*^Ter/Ter^ genotypes.

### Morphological analysis of bone marrow

To investigate whether the observed difference in hematological profile could be reflected by morphological changes in bone marrow, smears, and imprints of bone marrow from 3 months old *PRNP*^+/+^ (*n* = 1) and *PRNP*^Ter/Ter^ (*n* = 1) goat kids were analyzed. However, there were no differences in myeloid/erythroid ratio between the two genotypes, and the precursor cells had a normal appearance. In addition, there was no observable difference in degree of apoptosis (data not shown). Altogether, no evident morphological changes were found within the bone marrow of the *PRNP*^Ter/Ter^ goat kid.

### Monocyte phagocytosis and T-cell proliferation appear unaltered in *PRNP*^Ter/Ter^ animals

**Figure 4:**
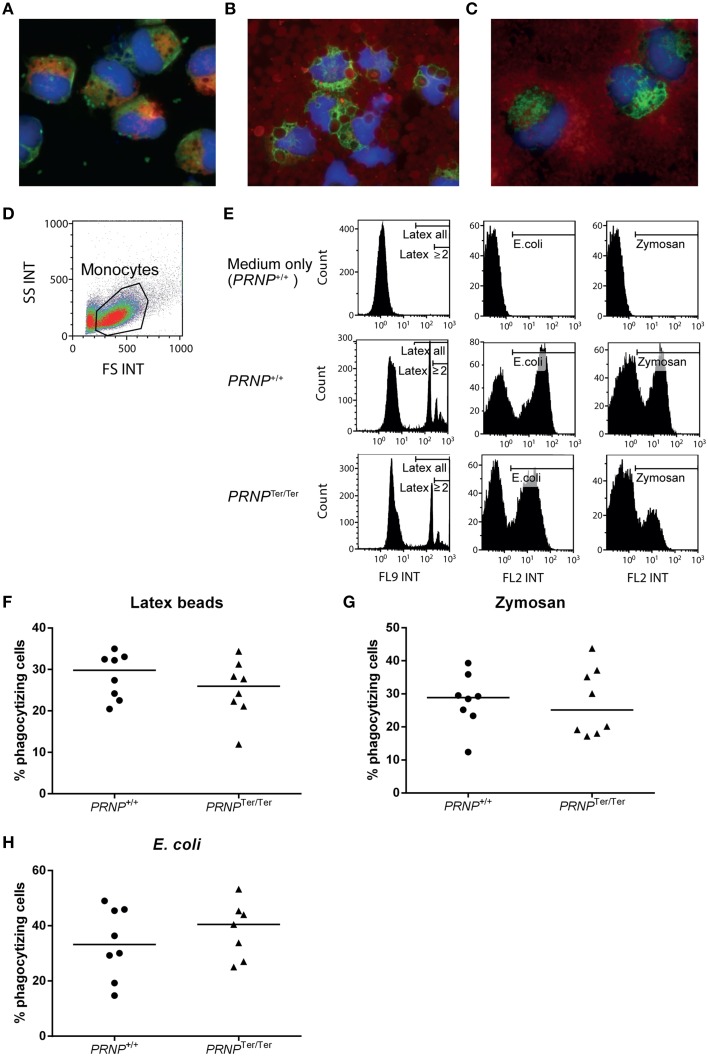
**Phagocytosis assays**. Cytospots confirmed the cellular uptake of **(A)** latex beads (green fluorescence), **(B)** *E. coli* (red fluorescence), and **(C)** Zymosan (red fluorescence) in activated monocytes. Additional staining for nuclei (blue) and CD68 (**A** red, **B** and **C** green). **(D)** Gating of live cells based on FS and SS characteristics in flow cytometry. **(E)** Particle uptake in *PRNP*^Ter/Ter^ and *PRNP*^+/+^ cells based on the results from two representative animals. Medium only was used as control. Gates indicate particle-containing cells, and in the case of latex beads, also gates for cells that had engulfed 2 particles or more **(F–H)** Compiled results of all animals showing percentage of monocytes containing **(F)** latex beads, **(G)** Zymosan and **(H)** *E. coli*, as measured by flow cytometry. For each assay, *n* = 8, except *PRNP*^Ter/Ter^ *E. coli* where *n* = 7.

To determine whether PrP^C^ might have a functional impact on white blood cells, we performed phagocytosis and proliferation studies to assess two major functional properties of leukocytes. Positively selected CD14^+^ monocytes from peripheral blood were cultured for 24 h to stabilize cells following isolation, and supplemented with GM-CSF to activate the cells and prevent apoptosis (Bratton et al., 1995). The resulting short-term activated monocytes were incubated with latex beads, bacteria (*Escherichia coli*), or zymosan-covered yeast cells (*Saccharomyces cerevisiae*) for 30 min. Fluorescence and confocal microscopy of cytospots confirmed the cellular uptake of particles (Figures 4A–C and data not shown). A majority of the monocytes were CD68^+^, consistent with monocytes or macrophages as previously described (Fadini et al., 2013). These cells had numerous vacuoles in the cytoplasm and a round to bean-shaped nucleus. All particle types were efficiently phagocytized by activated monocytes (Figures 4D,E). When comparing activated monocytes from *PRNP*^+/+^ and *PRNP*^Ter/Ter^ goats by flow cytometry, we detected no significant differences between the genotypes in the proportions of cells that had taken up fluorescent particles, for none of the particle types (Figures 4 F–H). There was also no significant difference in the numbers of particles per cell measured as median fluorescent intensity of positive cells; or in the case of latex beads, the number of cells that had engulfed 2 beads or more (Figure 4E and data not shown).

**Figure 5:**
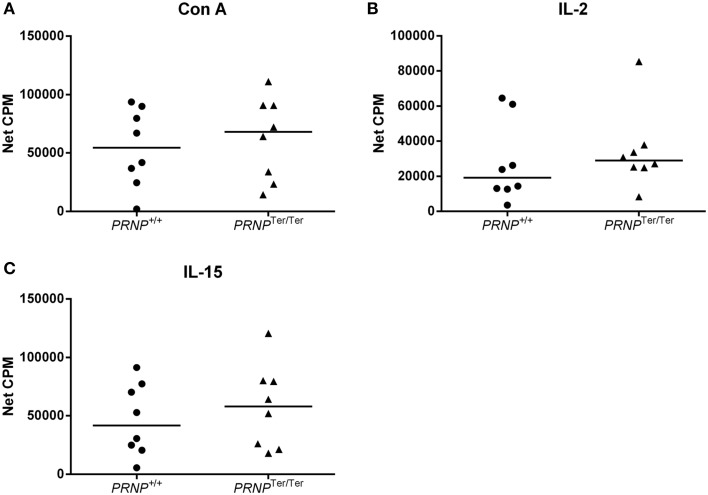
**Lymphocyte proliferation test**. Proliferation of T cells after 72 h stimulation with **(A)** Con A, **(B)** IL-2, and **(C)** IL-15, measured as beta emission following DNA incorporation of tritiated thymidine (net CPM = sample CPM − control CPM). In all cases *n* = 8.

To investigate if PrP^C^ expression could be involved in cellular proliferation, we stimulated PBMCs *in vitro* using the mitogen Con A or the cytokines IL-2 or IL-15 to cover proliferation of T cells and NK cells/innate lymphocytes (Figure 5). The cell cultures proliferated well in response to these stimuli, but no significant differences between the groups were observed, although a slightly higher median response of cells from the *PRNP*^Ter/Ter^ group was noted for all stimulations.

Collectively, these results suggest that loss of PrP^C^ does not have any major influence on the phagocytic ability of activated monocytes or the proliferative capacity of T or NK cells *in vitro*.

## Discussion

The PrP^C^ is phylogenetically conserved and widely present in vertebrates (Harrison et al., 2010), pointing to an essential role for the organism. Non-transgenic animals naturally devoid of PrP^C^, which has not been reported until recently (Benestad et al., 2012), could provide essential information regarding PrP^C^ physiology and function. In the present study we have observed that such *PRNP*^Ter/Ter^ goats present with hematological changes, although without morphological changes in the bone marrow or alterations in major immune parameters.

By investigating complete blood counts of 3–4 weeks old goat kids we demonstrated that *PRNP*^Ter/Ter^ animals had a significantly higher number of RBC compared to matched *PRNP*^+/+^ controls. Tendencies of changes in other hematological values were also observed, such as lowered MCV and increased amounts of neutrophil granulocytes. These results are strikingly similar to the hematological observations in 10 months old PrP^C^ KO cattle (Richt et al., 2007), which had increased numbers of RBC, WBC, and neutrophil granulocytes, and lowered MCV and MCH. The authors of the study questioned if the gene cassette used in the transgenic procedure rather than the absence of PrP^C^ *per se* could have caused the observed differences. Our findings in naturally mutated animals strengthen the likelihood that PrP^C^ loss may physiologically affect RBC parameters and neutrophil numbers in young ruminants. Preliminary hematology results from adult goats have revealed no differences between the genotypes in any of the hematological values (unpublished results).

Several experimental approaches have shown that phenotypes related to loss of PrP^C^ are only clearly evident under various stressful conditions, such as tissue damage, infection, or anemia (Zivny et al., 2008; Gourdain et al., 2012). Likewise, it could be speculated that the increased demand for cell proliferation in the bone marrow during growth represents a physiological stress which reveals an otherwise cryptic phenotype caused by loss of PrP^C^. Accordingly; a relatively subtle impact would not be observable in adult goats with lower bone marrow activity.

Mice lacking PrP^C^ have been reported to be in a chronic state of systemic iron deficiency, ascribed to inefficient uptake and transport into the blood stream, as well as inefficient uptake in various recipient cells, which could be rescued by the reintroduction of PrP^C^ (Singh et al., 2009). To rule out any impact of iron deficiency on the erythroid lineage we analyzed iron levels in all investigated animals. Although a smaller cell volume could be a result of an iron deficiency, we did not detect any difference in circulating iron levels that would support a state of systemic iron deficiency in *PRNP*^Ter/Ter^ animals.

Studies in PrP^C^ KO mice have suggested a possible influence of PrP^C^ on long-term hematopoietic stem cells (LT-HSCs), as these cells showed reduced regenerative capacity when undergoing serial transplantations (Zhang et al., 2006). However, before the interventions, mice were normal with respect to blood cell levels and presence of cell lineage profiles in the bone marrow. In the present study, we did not observe morphological evidence of bone marrow impairment, as apoptosis rate and myeloid/erythroid ratio was found similar in a *PRNP*^Ter/Ter^ animal compared to a normal control. More comprehensive and cell-specific investigations are however needed to clarify whether there are changes in bone marrow cellular composition in growing *PRNP*^Ter/Ter^ kids, and further studies are needed to reach a final conclusion regarding possible differences between genotypes with respect to stem cell phenotypes in animals, both under normal and stressful conditions.

We detected high PrP^C^ expression in peripheral blood mononuclear leukocytes but not granulocytes of *PRNP*^+/+^ animals, similar to previous reports that have led to a particular interest for PrP^C^ functions in immune cells, several of which have suggested PrP^C^ involvement in immune cell functions (Dodelet and Cashman, 1998; Barclay et al., 1999; Durig et al., 2000; Holada and Vostal, 2000; Herrmann et al., 2001; Halliday et al., 2005; Dassanayake et al., 2012). Hematological analysis suggested a higher count of neutrophil granulocytes and possibly monocytes in *PRNP*^Ter/Ter^ kids, while flow cytometric analyses did not confirm any difference in CD14^+^ monocyte numbers, and relative numbers of other major circulating mononuclear cell subsets also appeared unaffected by PrP^C^ loss. Similar results, including increased neutrophil counts, have been found in KO cattle, although a tendency of more numerous γδ T cells in a group of four PrP^C^ cattle compared to four WT cattle, was found (Richt et al., 2007). Few similar studies have been done in murine PrP^C^ KO models but in a double KO, namely PrP^C^ and Doppel KO mice, no alterations in the immune cell populations were noted (Genoud et al., 2004). Seen together these results indicate that mechanisms that regulate the levels of immune cells in the blood are largely independent of PrP^C^.

It has been shown that PrP^C^ is expressed in regulatory and memory T cells (Li et al., 2001), and that levels of PrP^C^ increase during T-cell proliferation (Cashman et al., 1990; Mabbott et al., 1997). Furthermore, T cells lacking PrP^C^ display reduced proliferation rates (Mabbott et al., 1997). Here, we stimulated PBMCs *in vitro* with mitogen or cytokines to assess alternative proliferation pathways but detected no difference in the proliferation rates between the genotypes. This correlates with the findings in transgenic cattle (Richt et al., 2007). Nevertheless, a slightly higher mean response in the *PRNP*^Ter/Ter^ group consistent through all stimulations could suggest that proliferative differences may be present in more restricted lymphocyte subsets. Furthermore, we quantified the uptake of bacteria, yeast and latex particles by activated blood monocytes in order to approach different activation pathways of phagocytosis (Flannagan et al., 2012). We did not find that PrP^C^ deficiency had any influence on the proportion or efficiency of monocytes to phagocytize any of these particle types. Earlier studies on phagocytosis have been performed with cells from PrP^C^ KO mice and have given conflicting results. In an *in vitro* study of phagocytosis of *E. coli* by bone marrow-derived macrophages, the activity was enhanced in cells from PrP^C^ KO mice (Wang et al., 2014). Conversely, macrophages from PrP^C^ KO mice were shown to phagocytize latex beads at a lower rate than macrophages from WT mice (Uraki et al., 2010). In another study, De Almeida et al. (2005) found that PrP^C^ is a negative modulator of phagocytosis, as peritoneal macrophages from PrP^C^ KO mice phagocytized apoptotic cells at a higher rate than cells from WT mice *in vitro* and *in vivo*, a result confirmed by two different mouse strains. The results from the latter study have later been linked to polymorphisms in PrP^C^ flanking genes involved in phagocytosis, rather than the loss of PrP^C^ in itself (Nuvolone et al., 2013), and similar concerns might be raised regarding other reports on PrP^C^ functions from PrP^C^ KO models. Our results from naturally PrP^C^-devoid animals are not in support of a principal mechanistic role of the prion protein neither in lymphocyte proliferation nor in phagocytosis.

In summary, this study showed that naturally occurring PrP^C^-deficient goat kids displayed a generally healthy constitution, moderate shifts in red blood cell and possibly granulocytic cell lineages but not in other major circulating hematopoietic cells. No impairment of major immune cell functions was detected. These results point toward a role for PrP^C^ related to maturation and release of selected cell lineages from the bone marrow, and underscore previous observations that PrP^C^ loss appears to have limited physiological or pathological consequences for the animal, at least under conventional living conditions and in the absence of significant stressors. The present natural goat model offers a unique opportunity to study the function of PrP^C^ *in* and *ex vivo* without the confounding factors of genetic engineering, which may hopefully help to shed light on the elusive nature of this protein and its related diseases.

## Author contributions

PB, AE, MT, MB, MR designed the study. MR, PB, MB performed the experiments and MR, PB, MT, AE, MB analyzed the data. AL assisted with the monocyte isolation protocol and experiments and HB contributed to the analysis of hematology data, blood smears and bone marrow imprints, and smears. MR, MB, HB, AL, IO, MT, AE, PB wrote or critically reviewed the manuscript.

### Conflict of interest statement

The authors declare that the research was conducted in the absence of any commercial or financial relationships that could be construed as a potential conflict of interest.
