# Supplementary material for: Hematological shift in goat kids naturally devoid of prion protein
Source: Front Cell Dev Biol. 2015 Jul 8;3:44. doi: 10.3389/fcell.2015.00044 (PMC4495340; doi:10.3389/fcell.2015.00044)
Supplement: Supplementary file 1 [file Table1.DOCX]

**Supplementary Table 1: Primary and secondary antibodies**

| Primary antibodies |  |  |  |  |  |  |  |
| --- | --- | --- | --- | --- | --- | --- | --- |
| Molecule recognized | **Monoclonal/Polyclonal** | **Clone** | **Isotype** | **Typical expression pattern** | **Manufacturer** | **Raised against** | **Used in (FCM = flow cytometry, CS = cytospots)** |
| B-B2 | M | BAQ44a | mouse IgM | B-cells | VMRD / Kingfisher Biotech | Bovine | FCM |
| CD14 | M | VPM65 | mouse IgG1 | Monocytes | Serotec | Ovine | FCM |
| CD14 | M | Tük4 | mouse IgG2a | Monocytes | AbD Serotec | Human | FCM |
| CD14 | M | CAM36a | mouse IgG1 | Monocytes | VMRD / Kingfisher Biotech | Bovine | FCM |
| CD3 | M | CD3-12 | ratIgG1 | T cells | AbD Serotec, Ltd., Oxford, UK | Human | FCM |
| CD4 | M | GC50A1 | mouse IgM | T cell subset | VMRD / Kingfisher Biotech | Bovine | FCM |
| CD68 | M | EBM11 | mouse IgG1 | Macrophages, monocytes | Dako | Human | CS |
| CD8a | M | BAQ111a | mouse IgM | T cell subset | VMRD / Kingfisher Biotech | Bovine | FCM |
| CD8b | M | BAT82a | mouse IgG1 | T cell subset | VMRD / Kingfisher Biotech | Bovine | FCM |
| FITC isotype control | M | eBRG1 | rat IgG1 | Unknown | Affymetrix / eBioscience |  | FCM |
| Pacific blue isotype control | M | MOPC-173 | rat IgG2a | Unknown | BioLegend |  | FCM |
| PrP^C^ | M | BAR224 | mouse IgG2a | Various | Bartin Pharma | Ovine | FCM |
| PrP^C^ | M | SAF32 | mouse IgG2b | Various | Bartin Pharma | Hamster | FCM |
| PrP^C^ | M | 6H4 | mouse IgG1 | Various | Prionics | Human | FCM |
| TCR1 | M | GB21a | mouse IgG2b | Gamma-delta T-cells | VMRD / Kingfisher Biotech | Bovine | FCM |
|  |  |  |  |  |  |  |  |
| Secondary antibodies |  |  |  |  |  |  |  |
| Fluorochrome | **Monoclonal/Polyclonal** | **Clone** | **Isotype recognized** |  | **Manufacturer** |  | **Used in** |
| Alexa 488 | P | (N/A) | Goat anti-mouse IgG1 |  | Life Technologies / Molecular Probes |  | FCM, CS |
| Alexa 488 | P | (N/A) | Goat anti-mouse IgG2a |  | Life Technologies / Molecular Probes |  | FCM |
| Alexa 488 | P | (N/A) | Goat anti-mouse IgG2b |  | Life Technologies / Molecular Probes |  | FCM |
| Alexa 488 | P | (N/A) | Goat anti-mouse IgG3 |  | Life Technologies / Molecular Probes |  | FCM |
| Alexa 488 | P | (N/A) | Goat anti-mouse IgM |  | Life Technologies / Molecular Probes |  | FCM |
| Alexa 594 | P | (N/A) | Goat anti-mouse IgG1 |  | Life Technologies / Molecular Probes |  | CS |
| PE | P | (N/A) | Goat anti-mouse IgG1 |  | Southern Biotechnologies |  | FCM |
| PE | P | (N/A) | Goat anti-mouse IgG2b |  | Southern Biotechnologies |  | FCM |
| PE | P | (N/A) | Goat anti-mouse IgG3 |  | Southern Biotechnologies |  | FCM |
| PerCp-efluor710 | M | R2a-21B2 | Rat anti-mouse IgG2a |  | Affymetrix / eBioscience |  | FCM |
| PerCp-efluor710 | M | M1-14D12 | Rat anti-mouse IgG1 |  | Affymetrix / eBioscience |  | FCM |
| Alexa 647 | P | (N/A) | Goat anti-mouse IgG2a |  | Life Technologies / Molecular Probes |  | FCM |
| Alexa 647 | P | (N/A) | Goat anti-mouse IgG1 |  | Life Technologies / Molecular Probes |  | FCM |
| Alexa 647 | P | (N/A) | Goat anti-mouse IgG2b |  | Life Technologies / Molecular Probes |  | FCM |
| APC-efluor780 | M | II/41 | Goat anti-mouse IgM |  | Affymetrix / eBioscience |  | FCM |
