# Supplementary material for: Hematological shift in goat kids naturally devoid of prion protein
Source: Front Cell Dev Biol. 2015 Jul 8;3:44. doi: 10.3389/fcell.2015.00044 (PMC4495340; doi:10.3389/fcell.2015.00044)
Supplement: Supplementary file 2 [file Table2.DOCX]

**Supplementary Table 2**

|  | Reference range | Median |  |  | P-values |  |  |
| --- | --- | --- | --- | --- | --- | --- | --- |
|  |  | A:  *PRNP^+/+^* | B:  *PRNP*^+/Ter^ | C:  *PRNP*^Ter/Ter^ | A vs. B | B vs. C | A vs. C |
| Aspartat aminotransferase (U/l) | 70-188 | 65 | 64 | 64 | 0,84 | 0,87 | 0,68 |
| Gamma-glutamyl transeferase (U/l) | 33-159 | 35 | 39 | 43,5 | 0,2 | 0,54 | 0,12 |
| Glutamate dehydrogenase (U/l) |  | 5,5 | 7 | 8 | 0,58 | 0,34 | 0,39 |
| Creatinin kinase (U/l) | 138-721 | 147,5 | 218,5 | 213,5 | 0,06 | 0,78 | 0,076 |
| Total protein (g/l) | 64-91 | 59 | 60 | 59 | 0,24 | 0,56 | 0,98 |
| Urea (mmol/l) | 2,7-11,9 | 3,5 | 3,4 | 2,95 | 0,56 | 0,5 | 0,43 |
| Creatine (µmol/l) | 61-120 | 60 | 61,5 | 64,5 | 0,6 | 0,23 | 0,049 |
| Total bilirubin (µmol/l) |  |  |  |  |  |  |  |
| Hydroxybutyric acid (mmol/l) | 0,2-0,8 | 0,1 | 0,1 | 0,1 | 0,08 | 0,16 | 0,62 |
| Glucose (mmol/l) | 2,7-4 | 6,2 | 6,05 | 6,25 | 0,81 | 0,46 | 0,35 |
| Inorganic phosphate (mmol/l) | 1,1-3 | 3,45 | 3,4 | 3,7 | 0,58 | 0,15 | 0,08 |
| Calsium (mmol/l) | 2,2-2,8 | 2,7 | 2,7 | 2,75 | 0,85 | 0,48 | 0,53 |
| Magnesium (mmol/l) | 0,79-1,22 | 1,085 | 1,045 | 0,985 | 0,15 | 0,27 | 0,005 |
| Albumin (g/l) | 31,8-44,5 | 32,4 | 32,85 | 33,65 | 0,25 | 0,92 | 0,62 |
| Alpha 1 (g/l) | 6-10,3 | 7,95 | 6,95 | 7,65 | 0,05 | 0,12 | 0,63 |
| Alpha 2 (g/l) | 5,2-8,9 | 8,25 | 8,55 | 8,5 | 0,11 | 0,94 | 0,09 |
| Beta 1 (g/l) | 0,8-4 | 0,9 | 0,9 | 1 | 0,78 | 0,22 | 0,25 |
| Beta 2 (g/l) | 2,6-5 | 2,65 | 2,65 | 2,35 | 0,81 | 0,37 | 0,29 |
| Gamma (g/l) | 12,5-31,3 | 6,3 | 7,75 | 5,95 | 0,14 | 0,22 | 0,42 |
| A/G | 0,59-1,28 | 1,25 | 1,22 | 1,25 | 0,77 | 0,78 | 0,91 |
| Fe (µmol/l) | 16-40 | 7 | 6,5 | 6 | 0,61 | 0,75 | 0,27 |
